# Correlation between pattern and mechanism of injury of free fall

**DOI:** 10.1007/s11751-012-0142-7

**Published:** 2012-10-06

**Authors:** Ismael Auñón-Martín, Pedro Caba Doussoux, Jose Luís León Baltasar, Elena Polentinos-Castro, Juan Pretell Mazzini, Carlos Resines Erasun

**Affiliations:** 1 “12 de Octubre” Hospital, Avenida de Cordoba s/n., 28041 Madrid, Spain; 2 Family Medicine Unit, North Area, Primary Care, Madrid Health Care System, Madrid, Spain; 3 Campbell Clinic Orthopaedics, Memphis, TN USA

**Keywords:** Free fall, Polytrauma, ISS, NISS, Mortality rate

## Abstract

To define the pattern of injury and aetiology of death of patients who have sustained major trauma due to high fall and its relationship with the mechanism of free fall. A total of 188 consecutive patients who sustained a high fall were included after the TRAUMASUR database was retrospectively reviewed. Demographic characteristics, severity scores, injury type, aetiology of high fall, mortality rate and aetiology of death were analysed. The mean age was 39.7 years (SD 15.5). The main aetiologies were work related (40.4 %) and suicide attempt (22.3 %). The mean injury severity score (ISS) and New Injury Severity Score (NISS) were 27.3 and 34.1, respectively. The most common cause of mortality within the intentional group was exsanguination (66 %), and the most frequent aetiology of death within the non-intentional group was endocranial hypertension (69 %). Differences were found with regard to the pattern of injuries and the aetiology of death according to the mechanism of free fall.

## Introduction

In 2007, mortality rates of 3.8/100,000 and 7.2/100,000 due to falls and suicides, respectively, were reported in Spain. There were 3,251 deaths as a result of suicides, with 696 cases due to free fall [1]. Fall from a height has been described as the most frequent mechanism of self-inflicted trauma [2].

Injuries from falls from heights represent a different type of blunt trauma; almost all of these patients have multiple injuries and the musculoskeletal system is frequently involved [3–7]. The severity of the injuries can be explained by physics with the velocity of impact calculated using the following formula: *v* = √2*gh*. The person at a height (*h*) above the ground is subjected to gravitational force (*g*) and its potential energy (Pe: *m* × *g* × *h*) is converted into kinetic energy (Ke: ½ × *m* × *v*^2^) in a fall. This means a great amount of energy is transferred to that patient.

Knowledge of injury patterns can help during the assessment of fall victims as they facilitate focus on specific areas. The characteristics of injuries and outcomes of vertical deceleration injuries depend on several factors: the level of the fall [5, 6, 8]; the landing position of the body; the surface onto which the victim falls [8, 9]; age; and comorbidities of the patient [8–10]. Knowledge of the injury patterns and the differences between those who jump and those who accidentally fall may help in the evaluation of these patients.

The aim of this study is to describe the patterns of injury in patients who fell from a height, the correlation between the mechanism of falling and pattern of injury as well as the aetiology of death, and the factors related to mortality rate.

## Materials and methods

The TRAUMASUR database was used to retrospectively identify the patients who had sustained a fall from height. A total of one hundred and eighty-eight patients were retrospectively reviewed and they form the basis of this report. TRAUMASUR is a prospective registry of patients with severe trauma due to different aetiologies from a level I trauma centre covering an area of almost 500,000 patients. Major trauma was defined as an injury with an injury severity score (ISS) ≥9 [11]. Patients who were excluded included those who died in the emergency care, were referred to another centre in the first 24 h, or had an ISS higher than 9 but due to low-energy mechanisms.

Demographic data such as age, sex, nationality, details of the aetiology, characteristics of the trauma and the height of the fall were obtained. Physiological data such as the AO-OTA classification of the orthopaedic injuries, scales of severity of trauma such as the Abbreviated Injury Score (AIS) [12], the ISS, the New Injury Severity Score (NISS) [13] and mortality rates were also included. The individual probability of survival (SP) was calculated using the Trauma-related Injury Severity Score (TRISS) [14]. The time from initial care until arrival at hospital, the time of stay at the intensive care unit (ICU) or as an inpatient were also collected. There was a group of patients who were initially treated in other centres and transferred to this hospital in which treatment given in the first 24 h was not accessible.

We recorded the characteristics of the different aetiological groups: suicidal attempt group also called the intentional group, work-related group also called unintentional group and an ‘other mechanism’ group. The intentional and unintentional groups were more homogeneous than the ‘other mechanism’ group; due to this fact, the first two groups were used for comparison purposes. The patients in the ‘other mechanism’ group were excluded.

Statistical analysis: Univariate analysis was performed for several variables in order to study the association between the different variables and mortality rate as well as for comparisons between the intentional and unintentional groups. Subsequently, multivariate analysis of logistic regression was conducted to study association of mortality and variables such as AIS, NISS, ISS, sex, age, height and aetiology (intentional vs. non-intentional). This logistic regression model included variables that were statistically significant (*p* < 0.05) in the univariate analysis, and in the final model, only variables that were statistically significant were included.

SPSS-15 software was used for data analysis.

## Results

A total of 188 patients were included. The mean age was 39.7 years (SD 15.5); there were 160 males (85 %) and 28 females (15 %). The mean height of fall was 6.7 m (SD 4.1). The mean ISS was 27.3, higher than the overall mean ISS of 23.2 in the TRAUMASUR database (*p* < 0.05). The mean NISS was 34.1 in comparison with a mean NISS of 29.5 within the TRAUMASUR database.

**Fig. 1:**
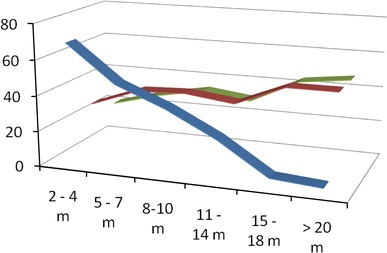
Correlation between the height of fall, number of patients and injury severity scores. *Light colour*: injury severity score (*ISS*). *Grey colour*: New Injury Severity Score (*NISS*). *Dark colour*: number of patients

A significant relationship between the height of free fall and injury severity (ISS and NISS) was found. This was not a linear relationship, but the higher the height of the fall, the higher the ISS (Fig. 1) and the lower the SP (individual probability of survival).

**Fig. 2:**
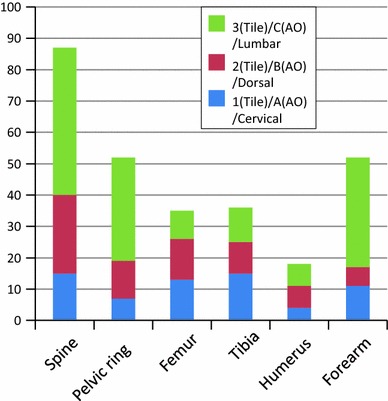
Orthopaedic injuries. *Light colour*: lumbar spine or tile C or AO 3. *Grey colour*: dorsal spine or tile B or AO 2. *Dark colour*: cervical spine or tile A or AO 1

With orthopaedic injuries, spinal fractures were the most commonly reported (87 cases). The lumbar spine was the most frequently involved (Fig. 2). Fifty-two pelvic ring fractures were identified, classified as type B or C (Tile classification [15]) in 45 patients (Table 1).

**Table 1:** Orthopaedic injuries associations

Orthopaedic injuries combination	Number of patients
Pelvic ring and long bone	28
Pelvic ring and long bone upper limbs	19
Pelvic ring and spinal fracture	15
Pelvic ring and long bone lower limbs	13
Pelvic ring and forearm	13
Tibia and forearm	12
Femur and tibia	12
Pelvic ring and tibia	11
Pelvic ring and humerus	10

The mean AIS for those with a head injury was 2.2. In 71 patients with head injury and who had an AIS >3, a mean ISS of 38.3 and SP of 0.58 were identified. A mortality rate of 31 % was recorded. The main cause of death in those patients with severe head injury was endocranial hypertension (ECH; 52 %).

There were 104 patients with lower limb trauma with a mean AIS of 1.6. Thirty patients (out of 104) had an AIS higher than 3; in this group, the mean ISS was 41 with a survival probability of 0.7 and a mortality rate of 30 %. The main cause of death in these patients was exsanguination (66 %).

The main causes of these falls from heights were either work-related accidents or attempted suicide; there were 76 cases (40.4 %) and 42 cases (22.3 %), respectively. A third group, ‘other mechanism’, had 70 cases (37.3 %) in which a more heterogeneous collection of mechanisms were identified, for example, sport-related, aggression or unknown.

The correlation between gender and aetiology of the fall was analysed. With male patients, work-related accidents were the main aetiology. No women were found within the work-related accidental group.

For the purpose of this study, aetiology was considered as unintentional (work related) and intentional (attempted suicide). Patients within the unintentional group tended to be older than the patients within the intentional group (*p* = 0.05); their injuries were sustained from falls from a lower height than patients within the intentional group. This difference was statistically significant (*p* < 0.01). The groups were also statistically different (*p* < 0.01) in their severity of injuries (ISS/SP, Table 2). No significant difference was found between the groups in the AIS for head injury. A higher AIS was observed within the attempted suicide group in comparison with the work-related one for those with lower limb injuries. There was a significant correlation between higher AIS in those with lower limb injuries and the mechanism of injury (intentional group; Table 2) and a significant correlation between a higher lower limb AIS and death by exsanguination.

**Table 2:** Characteristics of the two main aetiologic groups

	Work related (mean and SD or percentage)	Suicidal attempt (mean and SD or percentage)	*p*
Gender	100 % male, 0 % female	61 % male, 39 % female	0.000
Age	42 ± 12.5	37 ± 16.7	0.049
Height (m)	5.4 ± 2.8	10.1 ± 5	0.000
Exsanguination	15.4 %	66 %	0.000
Aetiology of death Exanguination			
Hic	69.2 %	0 %	0.000
Aetiology of death: ECH			
AIS head	2.4 ± 2.1	2.5 ± 1.9	0.86
AIS chest	1.7 ± 1.7	2.1 ± 1.9	0.24
AIS abd	0.69 ± 1.3	1 ± 1.6	0.22
AIS lower limbs	1 ± 1.5	2.7 ± 1.5	0.000
NISS	35.3 ± 18.1	38.3 ± 18.2	0.4
ISS	26.4 ± 13.3	35.1 ± 18.8	0.005
SP	0.83 ± 0.26	0.65 ± 0.33	0.002

*ECH* encocraneal hypertension

*p* < 0.05: Statistically significant

Twenty-seven deaths out of 188 patients were identified, representing an overall mortality rate of 14.3 %. Within this group, the mean age was 40 years (19–73). The mean fall was from a height of 8 m (3–18). The mean ISS and NISS were 45 and 57, respectively, and the mean SP 0.35. The two main aetiologies within the deceased group were intentional falls and work-related; the leading causes of death were endocraneal hypertension (ECH) and exanguination (Table 3). No statistically significant difference was found in the mortality rate of the intentional and unintentional group (Table 3). The ISS–NISS, PS and AIS head/neck were found to have statistically significant correlations with the mortality rate based on univariate analysis (*p* < 0.001, Table 4). An AIS head score ≥3 and ISS ≥15 were found to be independent factors correlated with mortality after the multivariate analysis was performed (*p* < 0.05).

**Table 3:** Characteristics of the two main aetiologic groups of deceased patients based on the aetiology

	Work related	Suicidal attempt	*p*
Number of patients (*n*)	64	42	
Number of deceased (*n*)	13	9	
Mortality	17 %	21.4 %	0.22
Mean height	5.7	9.8	0.005
ISS	43.8	53.1	0.005
SP	0.35	0.27	0.005
AIS head	4.6	3.9	0.2
AIS lower limbs	0.9	3.7	0.000
Aetiology of death	ECH (69 %)	Exsanguination (66 %)	0.000

*p* < 0.05: Statistically significant

**Table 4:** Univariate analysis of factors related to death

	Deaths (mean and SD or percentage)	Not deaths (mean and SD or percentage)	*p*
Gender	85 % male	85 % male	0.9
Age	40 ± 15.1	39.8 ± 15.5	0.9
Height (m)	7.7 ± 4.1	6.5 ± 4	0.18
Work related	12 %	88 %	0.25
Intentional	31 %	69 %	0.15
AIS head	4 ± 1.7	2 ± 2	0.000
AIS chest	2.2 ± 2	1.5 ± 1.7	0.051
AID abdominal	1.8 ± 1.5	0.64 ± 1.2	0.13
AIS lower limbs	2 ± 2.1	1.6 ± 1.6	0.3
ISS	45.3 ± 14.6	24.1 ± 12.5	0.000
NISS	57 ± 2.6	30 ± 1.1	0.000
PS	0.35 ± 0.3	0.88 ± 0.19	0.000

*p* < 0.05: Statistically significant

## Discussion

Falls from heights account for a significant percentage of urban trauma; these cases represent a distinctive mechanism of blunt trauma. It has been suggested that patients sustaining injuries from a fall from height have distinctive characteristics with an increased trauma severity and with different pattern of injuries in comparison with other mechanisms of blunt trauma [2–7, 16]. A fall from height is associated with the most severe blunt injuries as compared with a motor vehicle crash or other mechanisms [2]. Nguyen-Thanh et al. [16] reported significant differences in ISS and the distribution of fractures when a fall from a height was compared with a motor vehicle accident. This study supports that blunt trauma from falls from height is more severe than other mechanisms of trauma.

Patients who either attempt suicide or accidentally fall from a height usually present multiple injuries, with fractures being the most common injuries sustained [3–7]. Scalea et al. [3] reported 79 % of patients suffering from at least one major fracture and 60 % with multiple fractures. The most frequent sites were extremities (79 %), followed by spinal and pelvic fractures (24 and 23 %, respectively). Lowenstein et al. [4] also reported that the lower extremities were the most frequent areas involved in those patients. Velmahos et al. [5] reported skeletal injuries accounting for 76.2 % of all injuries with fractures of the extremities most frequently involved. Katz et al. [6] reported that there was no patient without a fracture of the extremity or spine. We found the skeletal system as the most commonly area sustaining injuries and spinal fractures as the most frequently identified. In spinal injuries, several studies have shown the lumbar spine and the thoracolumbar junction as the most frequently affected levels; this is also found in this study [3, 5, 6, 10, 16].

There is controversy in the literature over age as an independent predictor of death. We did not find a correlation between age and death, similar to the results reported by Chen-Chi Liu et al. [7]. In contrast, Demetriades et al. [10] found that the incidence of severe trauma and mortality rate increased significantly in those older than 65 years old. Lapostolle et al. [8] and David et al. [2] reported age was an independent predictor of death when a multivariate analysis was carried out on their samples.

The American College of Surgeons recommend that patients injured in falls from heights greater than 20 feet (1 m = 3.2 feet) need to be taken to a trauma centre. The relation between the height of the fall and the severity of injury is controversial. Helling et al. [17] found that an ISS >15 could be found in 35 % of patients who sustained injuries from a low fall; they also identified that low falls can be associated with life-threatening injuries even in patients who were stable initially. Goodacre et al. [9] reported that the height of fall as an isolated variable is a poor predictor of injury severity and did not identify a height threshold which can be used for trauma triage. Other authors found a positive correlation between height of fall and severity of injury [5, 18–20]. Our results support this correlation, but we are not able to find a height threshold which we could use to exclude significant injury (Table 1).

There is greater difficulty in establishing the correlation between the height of fall and mortality. Lapostolle et al. [8] reported a correlation between the height of fall and mortality. Chen-Chi Liu et al. [7] reported no statistically significant correlation between these variables as was also found in our study. We suggest that other variables such as the type of impact surface or contact preceding the impact may play a role in the final outcome; however, all of these factors are difficult to document accurately. None of these variables were studied in this review, this being a potential limitation of our study.

The mortality rate reported after falls from a height is quite variable (4.9–33 %) [2, 4, 5, 7, 8, 10]. This may be explained by different factors such as heterogeneity of the inclusion–exclusion criteria used, inclusion of the out-of-hospital mortality rate and severity of injuries amongst the studies. Lapostolle et al. [8] reported that 70 % of deaths were found in the out-of-hospital setting. We found a relatively high mortality rate of 14.3 % in this report and this could be explained as due to the severe trauma that presented in our patients with 77.6 % having an ISS ≥15. Some variables had been reported to be independent prognostic factors: age, height of the fall, body part first touching the ground, impact surface nature and AIS head/neck. In our study, only an ISS ≥15 and AIS head/neck ≥3 were found to be independent factors related to the mortality rate.

Intentional and non-intentional falls from heights have been reported previously. Richter et al. [18] found no significant differences in the pattern of injuries between both groups. However, Teh et al. [21] found differences between both groups regarding severity. David et al. [2] found that patients within the intentional group were older, presented with a higher ISS and had a higher mortality rate. The intentional group had more rib, pelvis and lower limbs fractures.

The number of females was higher than males within the intentional group, and these patients sustained falls from a higher altitude in comparison with the non-intentional group of patients. Severity scores (ISS, NISS, AIS lower limbs) were higher in this group of patients, exsanguination the most important cause of death.

In contrast to the group of patients mentioned above, the non-intentional group was comprised of male patients only. These patients tended to be older than the intentional group with lower severity scores (ISS, NISS) and a higher survival probability (Table 4); the main cause of death was extracranial haemorrhage from cranial trauma.

This study has some limitations; it is a retrospective study and the out-of-hospital mortality rate was not taken into account as well as variables like comorbidities of the patients and details of the surface of impact.

## Conclusion

The study reports on a group of patients who have fallen from a height and have three characteristics of interest: this cohort is a large number of patients; some carry very high injury severity scores; and they have all been managed homogeneously. We identified significant differences that were related to the aetiology of trauma. This was not reported in other studies. Patients who had accidental falls tended to have lower scores on severity scales but had more serious head injuries with the most common cause of death being intracranial hypertension. Patients who fall in an attempt at suicide also present with serious head injury, but the most frequent cause of death was exsanguination related to severe lower limb injuries.

## References

[1] Official numbers of population: municipal census. National institute for statistics, Spain. Available from http://www.ine.es/

[2] David JS, Gelas-Dore B, Inaba K (2007). Are patients with self-inflicted injuries more likely to die?. J Trauma.

[3] Scalea T, Goldstein A, Phillips T (1986). An analysis of 161 falls from a height: the ′jumper Syndrome′. J Trauma.

[4] Lowenstein SR, Yaron M, Carrero R (1989). Vertical trauma: injuries to patients who fall and land on their feet. Ann Emerg Med.

[5] Velmahos GC, Demetrios D, Theodorou D (1997). Patterns of injury in victims of urban free-falls. World J Surg.

[6] Katz K, Gonen N, Goldberg I (1988). Injuries in attempted suicide by jumping from a height. Injury.

[7] Chen-Chi L, Chien-Ying W, Hsin-Chin S (2009). Prognostic factors for mortality following falls from a height. Injury.

[8] Lapostolle F, Gere C, Borron SW (2005). Prognostic factors in victims of falls from a height. Crit Care Med.

[9] Goodacre S, Than M, Goyder EC (1999). Can the distance fallen predict serious injury after a fall from a height?. J Trauma.

[10] Demetriades D, Murray J, Brown C (2005). High-level falls: type and severity of injuries and survival outcome according to age. J Trauma.

[11] Baker SP, O’Neill B, Haddon W (1974). The injury severity score: a method for describing patients with multiple injuries and evaluating emergency care. J Trauma.

[12] Committee on Medical aspects of Automovile Safety (1970). Rating the severity of tissue damage: I. The abbreviated scale. JAMA.

[13] Osler T, Baker SP, Long W (1997). A modification of the injury severity score that both improves accuracy and simplifies scoring. J Trauma.

[14] Boyd CR, Tolson MA, Copes WS (1987). Evaluating trauma care: the TRISS method. J Trauma.

[15] Pennal GF, Tile M, Waddell JP (1980). Pelvic disruption: assessment and classification. Clin Orthop Relat Res.

[16] Nguyen-Thanh Q, Tresllet C (2003). Les polytraumatismes sont plus graves apres chute dune grande Hauteur quäpres accident de la voie publique. Ann Chir.

[17] Helling T, Watkins M, Evans L (1999). Low falls: an underappreciated mechanism of injury. J Trauma.

[18] Richter D, Hahn MP, Ostermann PA (1996). Vertical deceleration injuries: a comparative study of the injury patterns of 101 patients after accidentsl and intentional high falls. Injury.

[19] Steedman DJ (1989). Severity of free-fall injury. Injury.

[20] Moeller K, Letsch R (1997). Injury pattern in leaps from a window. A case analysis of 48 patients. Unfallchirurg.

[21] Teh J, Firth M, Sharma A (2003). Jumpers and fallers: a comparison of the distribution of skeletal injury. Clin Radiol.

